# Applications of nanotechnology in orthodontics: a comprehensive review of tooth movement, antibacterial properties, friction reduction, and corrosion resistance

**DOI:** 10.1186/s12938-024-01261-9

**Published:** 2024-07-25

**Authors:** Longwen He, Wenzhong Zhang, Junfeng Liu, Yuemei Pan, Simin Li, Yueqiang Xie

**Affiliations:** Stomatological Hospital, School of Stomatology, Southern Medical University, No. 366, South of Jiangnan Boulevard, Guangzhou, 510280 China

**Keywords:** Dentistry, Orthodontics, Nanotechnology in orthodontics, Orthodontic tooth movement, Antibacterial properties, Friction reduction, Corrosion resistance

## Abstract

Nanotechnology has contributed important innovations to medicine and dentistry, and has also offered various applications to the field of orthodontics. Intraoral appliances must function in a complex environment that includes digestive enzymes, a diverse microbiome, mechanical stress, and fluctuations of pH and temperature. Nanotechnology can improve the performance of orthodontic brackets and archwires by reducing friction, inhibiting bacterial growth and biofilm formation, optimizing tooth remineralization, improving corrosion resistance and biocompatibility of metal substrates, and accelerating or decelerating orthodontic tooth movement through the application of novel nanocoatings, nanoelectromechanical systems, and nanorobots. This comprehensive review systematically explores the orthodontic applications of nanotechnology, particularly its impacts on tooth movement, antibacterial activity, friction reduction, and corrosion resistance. A search across PubMed, the Web of Science Core Collection, and Google Scholar yielded 261 papers, of which 28 met our inclusion criteria. These selected studies highlight the significant benefits of nanotechnology in orthodontic devices. Recent clinical trials demonstrate that advancements brought by nanotechnology may facilitate the future delivery of more effective and comfortable orthodontic care.

## Introduction

**Fig. 1:**
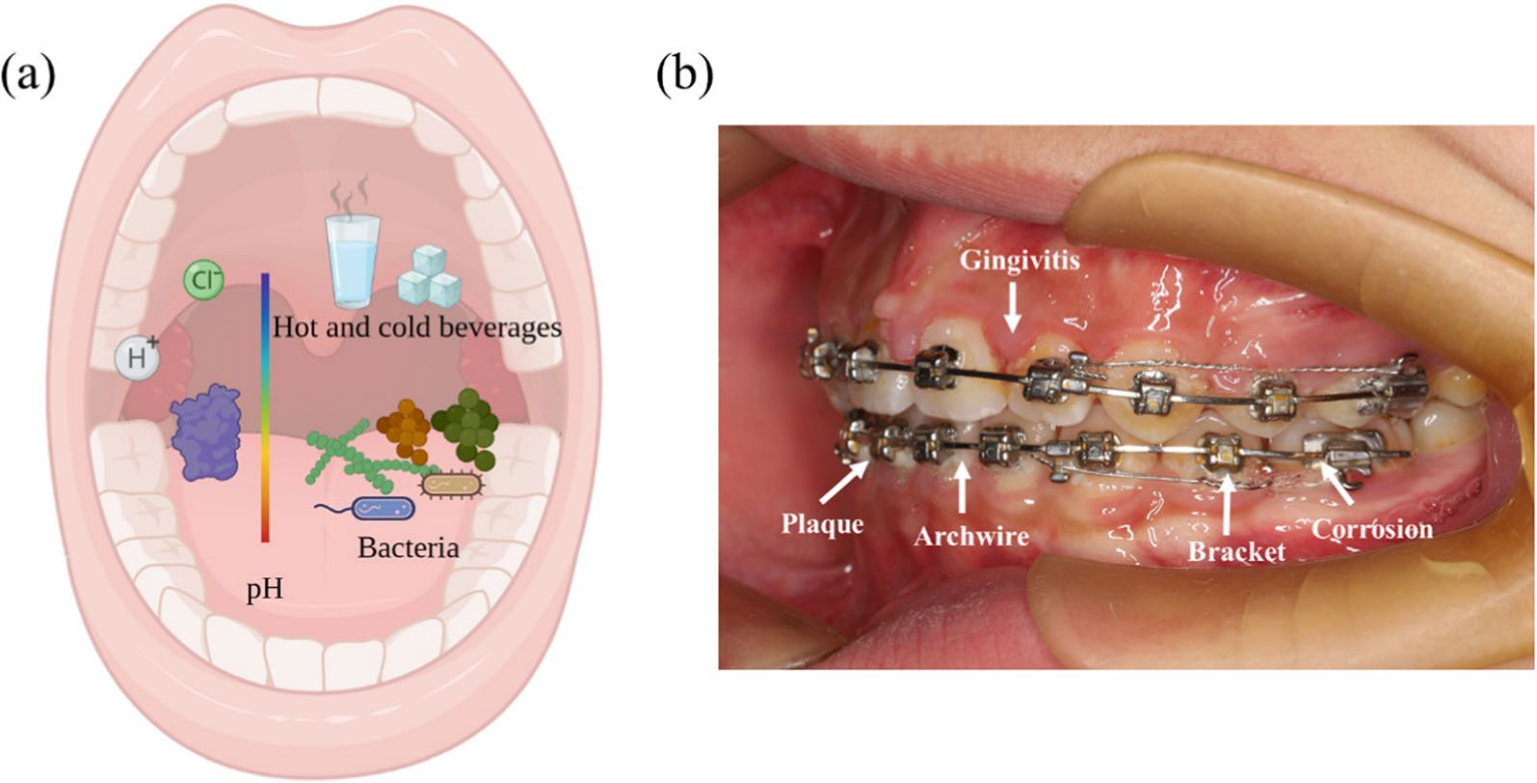
**a** Complex oral environment; **b** challenges faced during traditional fixed orthodontic therapy include plaque accumulation, gingivitis, appliance corrosion, and metal ion precipitation

Malocclusion is the misalignment or improper spatial relationship between the teeth of the two dental arches [1]. It is among the most prevalent dental disorders worldwide. The World Health Organization estimates that 60–75% of the global population is afflicted by various types of malocclusion (crowded teeth, overbite, underbite, crossbite, diastemas, etc.) [2]. Timely orthodontic treatment is crucial to correct malocclusion and prevent associated complications. An essential component of treatment is the use of orthodontic materials, such as auxiliary devices and fixed and removable appliances. However, the oral environment is complex, and the potential complications of the use of these materials remain unresolved (Fig. 1). Orthodontic practice is beset by numerous challenges. Novel solutions are required to facilitate the efficient movement of teeth; improve alveolar bone remodeling and prevent black triangles; reduce biofilm formation on instruments and auxiliary equipment; decrease tooth surface demineralization and cariogenesis; and avoid metal corrosion in traditional fixed orthodontic devices.

Nanotechnology encompasses the use of minute machinery that can manipulate matter on an extremely small scale. Nanotechnology has been widely used for biomedical purposes that range from diagnosis and treatment to the modification of medical devices and the facilitation of personalized health care [3–5]. Nanomaterials, which have dimensions between 1 and 100 nm, have generated interest in the field of regenerative medicine because of their distinctive optical, mechanical, magnetic, electrical, and catalytic properties [6]; which also account for their excellent immunological evasion, permeability, and tunability. As such, they offer great promise for tissue engineering [7], antimicrobial therapy [8], drug delivery [9], and functional imaging (MRI and CT) [10].

Nanodentistry is the application of such technology to dental care [11, 12]. Dental professionals and researchers have already made significant progress that has been facilitated by advances in nanomaterials, nanorobots, and nanoengineering [13, 14]. Nanotechnology is used in a plethora of newly developed dental products ranging from implants to mouthwashes, and its integration into orthodontics is already underway.

**Fig. 2:**
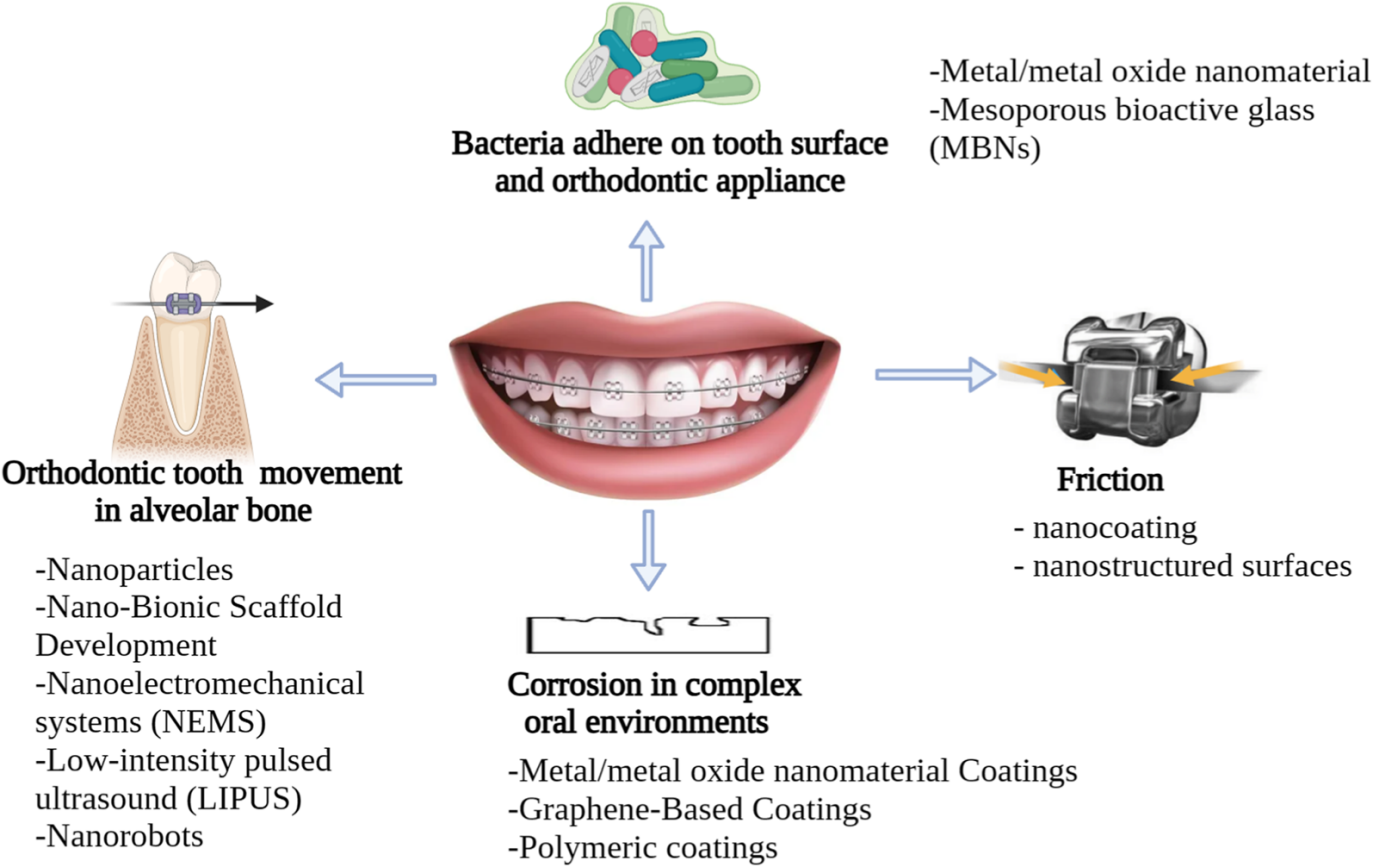
Schematic summary of the current review work

This review focuses on the use of nanotechnology to control orthodontic tooth movement (OTM) and improve alveolar bone repair, as well as to prevent biofilm formation and demineralized lesions of the enamel, referred to as white spot lesions (WSLs) (also known as the scars of orthodontic treatment). Nanocoating of wires and brackets is performed to increase the effectiveness of brackets and decrease friction on archwires used in traditional orthodontic treatment, and to increase safety and biocompatibility by resisting corrosion and minimizing the precipitation of hazardous materials. Additionally, this review explores potential future orthodontic applications of nanotechnology (Fig. 2).

## Methods

### Literature search strategy

Databases such as PubMed, Web of Science Core Collection, and Google Scholar were used for the literature search in December 2023. Search terms were: ((‘nanotechnology’ OR ‘nanomaterial’ OR ‘nanoparticle’ OR ‘nanostructure’) AND (‘orthodontics’ OR ‘dentistry’)) AND ((‘tooth movement’ OR ‘orthodontic movement’) OR ‘antibacterial’ OR ‘friction reduction’ OR (‘corrosion resistance’ OR ‘anticorrosion’)) NOT review. The search process also included manual searching. Subsequently, studies were evaluated for their eligibility.

### Literature screening and selection criteria

The study selection and qualitative analysis were performed independently by two reviewers (LWH and SML) using the Preferred Reporting Items for Systematic Reviews and Meta-Analyses (PRISMA) guidelines. The titles and abstracts of the publications identified by the databases were screened, and the reference lists of critical articles were hand-searched for relevant articles. The full texts were examined during the second stage to determine whether the articles met the selection criteria.

Inclusion criteria were: (1) application of nanomaterials to enhance OTM, antibacterial activity, corrosion resistance, or friction reduction, thereby improving the efficiency of tooth movement; (2) eligible studies could include physicochemical research and also biomedical studies testing nanomaterials in cellular and/or animal models.

Exclusion criteria were: (1) studies of non-nanomaterials; (2) applications of nanomaterials not designed to enhance tooth movement, antibacterial properties, corrosion resistance, or friction reduction, but for other dental disciplines such as periodontology or implantology; (3) non-SCI (Science Citation Index) papers were not considered.

## Results

**Fig. 3:**
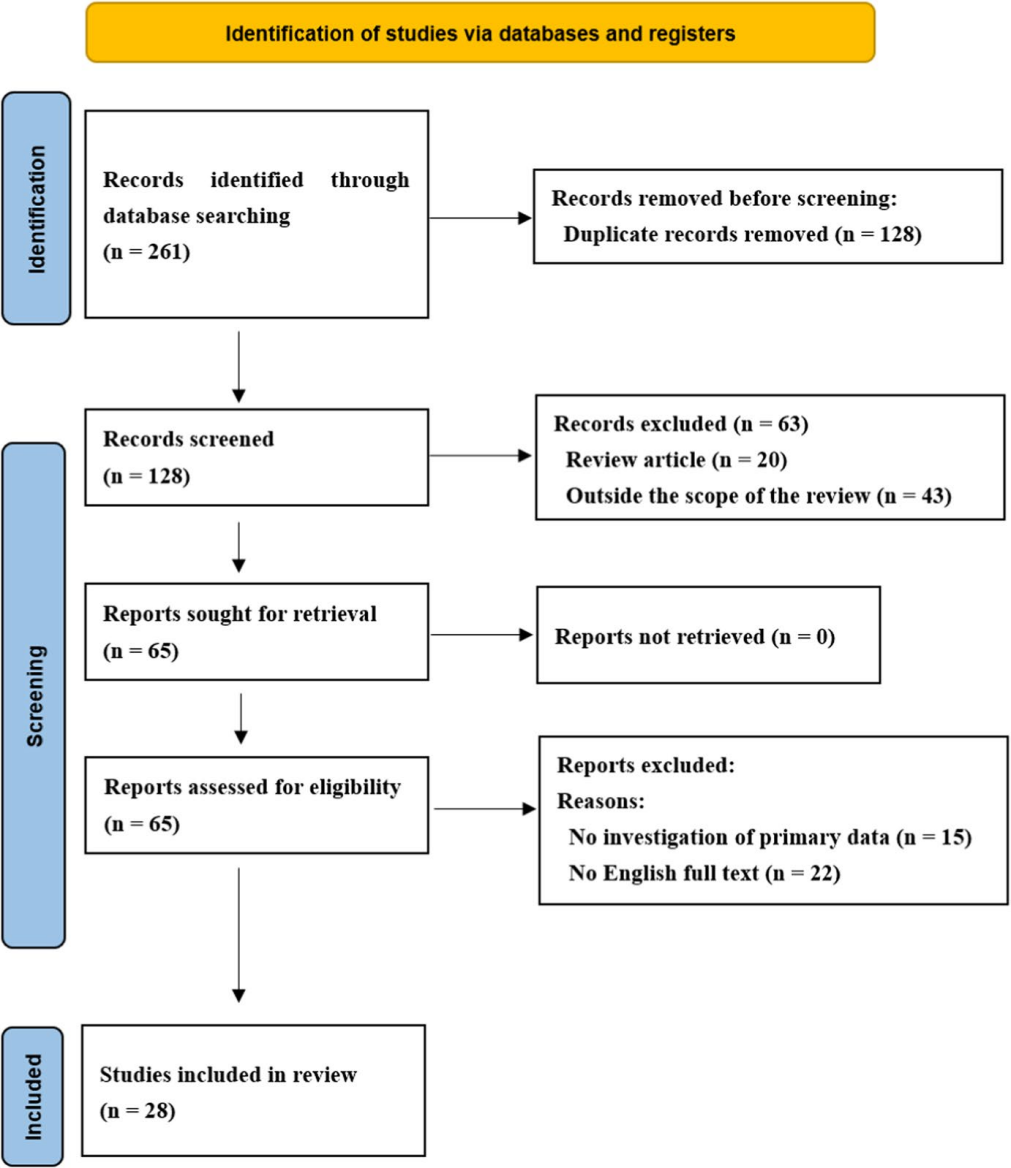
Representation of selection of articles through PRISMA framework

The results obtained by adhering to PRISMA guidelines are depicted in Fig. 3. Systematic and manual searches yielded 261 studies. After screening, 28 studies met eligibility criteria and were included in this study.

## Discussion

### Controlled orthodontic tooth movement (OTM)

The durations of current orthodontic regimens are prolonged. A typical treatment course requires roughly 2 to 3 years, which ultimately jeopardizes patient compliance. Long-term orthodontic therapy predisposes patients to iatrogenic complications such as WSLs [15], caries, gingivitis [16], and root resorption. The enormous demand for shorter orthodontic treatment durations has led to a general interest in research focused on abbreviating the time spans required for OTM. By accelerating bone remodeling, the duration of orthodontic treatment could be significantly reduced. A great deal of research has been focused on hastening OTM by investigating surgical and nonsurgical interventions such as corticotomy [17], distraction osteogenesis [18], and the new research hotspot of nanotechnology [2, 19].

Furthermore, in some circumstances, slower rather than more rapid rates of OTM may be preferred to prevent unintended anchoring loss and post-treatment relapses. An alternative to all of these techniques is the development of novel biomaterials and innovative systems for the delivery of bioactive molecules such as growth factors and hormones that have been administered locally to paradental tissues in animal models to stimulate or inhibit the rate of OTM [20, 21] Advances in nanotechnology have generated interest in the application of nanomaterials to accelerate or decelerate OTM.

#### Nanoparticles

OTM is attributed to mechanical stimulation and subsequent proliferation of alveolar bone and periodontal ligament (PDL). The regulation of OTM involves alterations of tissue perfusion and levels of inflammatory cytokines [20]; growth factors [22]; neurotransmitters and growth of bioreactive substances of PDL; acid stimulating factors; and arachidonic acid products [23]. Because of their minute size and high surface area-to-volume ratio, nanoparticles (NPs) have the potential to regulate the physiology of cells involved in bone formation and absorption, thereby potentially accelerating or decelerating the rate of OTM. The acceleration of bone remodeling and OTM is induced by the promotion of osteoclastogenesis and angiogenesis through the stimulation of bone marrow mesenchymal stem cells (BMSCs) by reduced graphene oxide (GO) NPs. Furthermore, an analysis of mechanisms of action revealed the significant regulatory function of the PERK pathway in this particular process [24].

The long-term effects of nitric oxide (NO) on OTM were investigated in a rat model. NO-releasing silica NPs were injected locally. NO released from *S*-nitrosothiol-containing NPs inhibited tooth movement for 1 week post-injection. The inhibition of tooth movement by NO-releasing nanoparticles may be due to increased perfusion and consequent tissue oxygenation. This effect reduces local hypoxia induced during orthodontic tooth movement, thus reducing downstream signal induction and decreasing orthodontic tooth movement [25]. A possible explanation for NP-mediated acceleration or deceleration of OTM is that they promote non-mineralized reactions that can also increase or decrease osteogenesis or PDL remodeling. These reactions involve neovascularization and reorganization of nerve fibers in PDL.

#### Nano-bionic scaffold development

Tooth crowding, one of the most prevalent issues in orthodontics, is often treated by extractions to create more room in dental arches. Post-extraction ridge resorption and gingival ingrowth might encumber OTM because adequate bone volume is necessary to provide the intended outcome [26]; consequently, ridge augmentation may be indicated prior to tooth implant placement into the affected site. Ridge augmentation is also indicated for the treatment of other disorders including cleft palate and periodontal disease. Because of its osteogenic properties and biocompatibility, autogenous bone is the preferred graft material for repairing bony defects [26]. Materials containing calcium phosphate are frequently employed in bone tissue engineering and clinical medicine because their chemical compositions and biological characteristics are remarkably comparable to those of inorganic components of the human body [27, 28]. Calcium phosphate polymer-induced liquid precursors may be utilized for the biomineralization of craniofacial bone [29]. Nanoscale hydroxyapatite platelets, with a thickness of 2–4 nm, penetrate and enshrine type-I collagen fibrils, providing bones the necessary rigidity and strength to endure varying mechanical stresses. Calcium phosphate improves the efficacy of orthodontic treatment by extending the durability of regenerated bone surrounding shifted teeth and prolonging the viability of repaired periodontal tissues. Biomimetic growth factor-loaded triphasic scaffolds (GFSs) may closely resemble the natural structures of cementum, PDL and alveolar bone. GFSs exhibit minimal in vitro cytotoxicity, excellent biocompatibility, and good mechanical qualities. In periodontal ligament stem cells, each compartment of the structure containing indicator GFSs can stimulate the expression of genes linked to osteogenesis, growth of the periodontal ligament, and cementation. By enhancing the development of healthy periodontal tissue, biomimetic scaffolds loaded with GFSs also facilitated the repair of periodontal defects in a rat model [30].

#### Nanoelectromechanical systems (NEMS)

The basic structure of nanoelectromechanical systems (NEMS) typically comprises nanoscale mechanical sensing elements such as nanobeams, nanofilms, and nanotubes, coupled with electronic components such as electrodes, amplification circuits, and signal processing circuits [31]. The mechanical components respond to external physical quantities such as force, pressure, mass, and displacement by deforming. These deformations are then converted into measurable electrical signals by the electronic components. NEMS combine mechanical and electrical functions at the nanoscale [31]. Compared with traditional microelectromechanical systems, NEMS offer higher sensitivity and resolution. OTM may benefit from the use of microfabricated biocatalytic fuel cells, also known as enzyme batteries, to produce electricity [32]. Glucose and other organic substances are mostly used by this process. An enzymatic micro-battery applied to the gingiva in close proximity to alveolar bone could provide an electrical power source to accelerate OTM [33]. Nanostructures can maximize enzymatic reactions because of their enormous surface areas [34]. These devices have the potential to enhance OTM by utilizing electricity to supplement mechanical forces [35–37]. NEMS-based systems may offer the best solutions to improve soft tissue biocompatibility and reduce the impact of food with variable temperatures and pH levels on the functionality of a microfabricated protein battery [32, 38]. In vivo studies revealed that 15–20 microamperes of low direct current delivered to the alveolar bone by bioelectric potential modification elevated the concentrations of the second messengers cAMP and cGMP in osteoblasts and periodontal ligament cells. These results demonstrated that electrical stimulation can increase cellular enzyme phosphorylation and trigger secretory and synthetic processes linked to faster bone remodeling, which can expedite the shifting of teeth into orthodontic alignment [39, 40]. Many nanostructured materials, including mesoporous media, NPs, nanofibers, and nanotubes, are effective hosts of enzyme immobilization. Orthodontic researchers are exploring the use of NEMS sensors to precisely measure orthodontic forces. This innovation aims to enable real-time monitoring and feedback control of these forces. The goal is to develop a smart orthodontic device that seamlessly integrates the application and monitoring of orthodontic forces through advanced miniaturization designs. However, the concept is primarily in the design and simulation phase and has not yet progressed to mature device development or clinical validation.

#### Low-intensity pulsed ultrasound (LIPUS)

Low-intensity pulsed ultrasound (LIPUS) improves cellular metabolism. It has attained regulatory approvals due to its proven efficacy in promoting bone regeneration and facilitating the healing of fractures. LIPUS significantly enhanced the distance of OTM and upregulated BMP-2 signaling in a rat model by activating the HGF/Runx2/BMP-2 signaling pathway and RANKL expression, thereby accelerating alveolar bone remodeling [41]. Starting from day 5, LIPUS significantly accelerated OTM and the activation of related signaling pathways, showing a clear difference compared with the control group [41]. LIPUS can also reorganize the cytoskeleton and affect fiber distribution [42]. In addition, LIPUS promotes osteoblast–osteoclast interaction through EphrinB2/EphB4 signaling, activating the EphB4 receptor on the osteoblast membrane. This transduces LIPUS-related mechanical signals into the cell, subsequently influencing the nuclear translocation of Yes-associated protein in the Hippo signaling pathway, thereby regulating cell migration and osteogenic differentiation [43]. The use of functionalized microbubbles in conjunction with ultrasound can augment the benefits of LIPUS by causing controlled mechanical stress and localized shear forces on cells. Nanobubbles, or lipid bubbles on a nanoscale, are very stable and biosafe. Cyclic arginine-glycine-aspartic acid-modified nanobubbles with particle sizes of ~ 500 nm may enhance LIPUS-induced osteogenic activity of BMSCs by acting as nanomechanical force generators through integrin receptors, the actin cytoskeleton, and intracellular calcium oscillations [44]. The addition of LIPUS to Invisalign SmartTrack® transparent aligners resulted in a 49% reduction of treatment duration and a roughly 66% increase in patient compliance [45]. In a prospective clinical trial, LIPUS increased the rate of OTM by an average of 29% and also conferred a statistically significant decrease in orthodontic root resorption (0.0092 ± 0.022 mm/week compared to 0.0223 ± 0.022 mm/week on the control side) [46].

#### Nanorobots

Nanorobotics is the field of designing and constructing nanorobots, whose components are at or near the nanometer scale. Nanorobots have been used to accelerate OTM through the application of NEMS and nano-LIPUS devices [47]. Nanorobots facilitate rapid and painless tooth movement within a few hours by directly manipulating the periodontium, including alveolar bone and the periodontal ligament. Additionally, nanorobotic toothpastes are administered once daily to cleanse both supra- and subgingival dental surfaces effectively, removing any debris and associated substances, while detecting biofilm-associated cariogenic bacteria [48]. OTM can be expedited by the application of electrical current or ultrasonic waves that stimulate cellular enzymatic phosphorylation and fibroblast growth factor release from a macrophage-like cell line (U937) [49]. Furthermore, these techniques offer the potential for tailoring wear time recommendations for patients with removable appliances, leading to a more effective, expedited, and comfortable orthodontic treatment [50–52].

In summary, although nanorobots present a promising tool for accelerating or decelerating OTM, more research and clinical trials are needed to fully understand their potential and to ensure their safety and efficacy in dental and orthodontic applications.

### Anti-microbial activity and tooth remineralization

Permanent colonization and increased plaque formation on orthodontic instruments and auxiliary devices constitute significant sequelae of orthodontic treatment, and predispose patients to complications, such as gingivitis and periodontitis. Additionally, the prolonged retention of dental plaque and adhesion of biofilms on these appliances disrupt the delicate equilibrium between demineralization and remineralization, resulting in WSLs.

Multiple reports have indicated that NPs possess commendable antibacterial properties [53]. The antibacterial efficacy of these nanostructured agents is ascribed to their substantial surface area that facilitates increased atom exposure on adjacent surfaces and thereby maximizes activity [54, 55], and their localization in close proximity to bacterial membranes. Additionally, their diminutive size facilitates their penetration across bacterial cell membranes [56, 57]. Consequently, the addition of antimicrobial NPs to orthodontic appliances is considered one of the most potentially effective strategies to improve patient outcomes [58, 59].

#### Metal/metal oxide nanomaterials

NPs that contain metals and metal oxides such as silver (Ag) [60, 61], zinc oxide (ZnO) [62], copper (Cu) [63], copper oxide (CuO) [64], magnesium oxide (MgO), and titanium dioxide (TiO_2_) [65] have been evaluated for their abilities to reduce bacterial colonization and dental plaque formation around orthodontic appliances. Electrostatic interactions play a crucial role in antibacterial activity by attracting positively charged metallic NPs to negatively charged bacterial cell membranes. Furthermore, their nanoscale size promotes the release of metal ions [56], which exert bactericidal activity by interacting with thiol groups of proteins and with DNA, thereby denaturing proteins, disrupting DNA unwinding, dysregulating metabolism, and inhibiting cell membrane synthesis, leading to perforation of bacterial cell membranes and cell death [8, 66, 67].

Metal/metal oxide NPs can produce reactive oxygen species (ROS) [68] such as hydroxyl radicals, hydrogen peroxide, and superoxide anions. ROS can damage cellular components such as DNA, proteins, and lipids, leading to bacterial cell death. Ag-generated free radical production has been demonstrated through electron spin resonance (ESR) analysis of Ag NPs [69, 70]. This confirms the relationship between the bactericidal activity of Ag NPs, free radical formation, and membrane damage induced by these radicals. Similarly, TiO_2_ NPs can generate potent bactericidal hydroxyl radicals [71].

Metal/metal oxide NPs may also exert bactericidal activity through non-oxidative mechanisms. A study demonstrated that the activity of three types of MgO NPs against *Escherichia coli* [72] was independent of oxidative stress, and proposed several mechanisms to support their findings. Firstly, the presence of pores in bacterial cell membranes suggested MgO NP-induced perforation. Notably, neither MgO NPs nor magnesium ions were observed within the bacteria. Secondly, intracellular ROS levels were low following MgO NP exposure. Thirdly, MgO NP exposure did not induce lipid peroxidation. Finally, levels of intracellular protein related to ROS were unaffected; however, metal/metal oxide NPs may impede several protein-associated metabolic pathways including amino acid, nucleotide, and carbohydrate metabolism [72].

Additionally, the disruption of bacterial metabolism by metal/metal oxide NPs impedes biofilm formation, which is a critical etiologic factor of dental disease. NPs adhere to and permeate biofilms, thereby affecting ion channels that facilitate long-distance interbacterial electrical signaling within the biofilm. This disruption influences membrane potentials, thereby promoting lipid peroxidation and DNA binding. Consequently, bacterial metabolism is dysregulated, leading to a reduction of biofilm synthesis [73–75]. Moreover, metal/metal oxide NPs may alter the surfaces of dental materials, making them less conducive for biofilm adhesion [76]. Orthodontic brackets coated with nanosilver exhibited smoother surfaces that displayed decreased adherence of *Streptococcus mutans* and *Streptococcus sobrinus* [67].

#### Mesoporous bioactive glass

Bioactive glasses release calcium and phosphorus ions and act as a source of various ions (SiO_2_, CaO, Na_2_O, and P_2_O_5_). The ions released earlier from bioactive glasses act as buffers, which increase the pH of the dissolution medium and prevent demineralization of the enamel. They also promote remineralization by facilitating hydroxyapatite formation. In particular, mesoporous bioactive glass NPs may load other biomolecules, and demonstrate potent bioactivity and antibacterial properties [77, 78]. GO with a bioactive glass mixture was added to orthodontic adhesive in different ratios, and showed potent antibacterial and anti-demineralization effects. GO exerts its antibacterial activity via two main mechanisms: deposition on the bacterial membrane, which introduces mechanical stress and isolates the bacterial cell surface from the environment, thus blocking membrane activity sites; and by generating oxidative stress [79, 80].

Calcium phosphate, as one of the important components of enamel, can be utilized in remineralizing agents to repair demineralization that may occur during orthodontic treatment [81, 82]. Remineralizing agents act in plaque and on tooth surfaces as reservoirs of Ca and P ions that can be released during an acidic cariogenic challenge to prevent demineralization and facilitate remineralization [83]. Fluoride-doped amorphous calcium phosphate NPs may carry materials with enhanced anti-cariogenic and remineralizing properties [84].

Moreover, mesoporous bioactive glass can synergize with metal NPs to improve performance [85, 86]. A sealant containing mesoporous glass-Ag NPs featuring a high specific surface area promoted remineralization by facilitating the penetration of dentinal tubules by hydroxyapatite crystals and achieved an excellent occlusion rate. Furthermore, the addition of YAG laser treatment inhibited the growth of *S. mutans* [87].

Notably, advanced nanotechnology was used to simulate the natural biomineralization process and synthesize dental enamel. Hydroxyapatite nanorods were synthesized and modified by adding surfactant monolayers that enabled self-assembly into enamel prism-like structures [88].

In summary, the use of nanotechnology to inhibit bacterial growth and biofilm formation and to facilitate dental remineralization offers a promising approach to enhance the care of patients receiving orthodontic therapy. However, the safety and potential toxicity of NPs are of crucial importance, especially because they are used in the oral cavity. Ongoing research and strict regulatory oversight are essential to ensure their safe use in dental and orthodontic applications.

### Nanotechnology reduces friction along archwires

Orthodontic treatment involves the sliding of a tooth along an archwire. This process generates a frictional force between the archwire and bracket, which is opposed to the movement itself [89]. Consequently, orthodontic force must exceed this resistance. More than 60% of orthodontic force applied to obtain OTM may be lost to friction, reducing the force employed by the fixed appliance. Friction reduction would allow the application of a lower orthodontic force and bring significant benefits, ranging from a lower risk of root resorption to optimal anchorage control and reduction of treatment duration [90].

#### Nanocoatings

Archwires can be coated with NPs or nanocomposite materials. These coatings are designed to be ultra-smooth and durable to significantly reduce the friction between the wire and the brackets. Materials such as titanium dioxide [91], silicon dioxide [92], graphene sheets [93, 94], and carbon nanotubes [95] are often used for these coatings. In addition to reducing WSL and caries, ZnO-NPs also reduce the friction coefficient of NiTi wires [96]. Moreover, some nanocoatings are self-lubricating. They release lubricant molecules gradually, maintaining a low-friction interface between the wire and the brackets over time. Lubricant polymers containing mineral NPs of boron nitride [95], inorganic fullerene-like tungsten disulfide [97], molybdenum disulfide [98], or certain ceramics [99] can be applied as thin films onto archwire surfaces. The NPs act as microscopic ball bearings to reduce friction between the sliding surfaces.

#### Nanostructured surfaces

Nanotechnology enables the creation of new alloys at the nanoscale level, which can be optimized for reduced friction and improved mechanical performance. A fractal structure featuring micro-domains with identical nanometer-sized grooves was assembled on the surfaces of orthodontic wires by using an oxygen plasma and acid corrosion [100]. The concave groove surfaces were dominated by titanium and convex segments were made of the same material as the bulk wires. The micro-nano fractal structure generated a hydrophobic surface with the largest contact angle to water being about 157°. The titanium-dominated nanolayer and the hydrophobicity of the surface vastly improved the corrosion resistance of orthodontic wire. The fractal structures of the wires self-assembled when they were immersed in acidic environment; the self-protection of the oxygen plasma-treated orthodontic wires in an acidic environment indicates their suitability for orthodontic applications. Nanotechnology can enhance the flexibility and strength of the archwires, enabling the application of gentler and consistent force. This reduces the stress and wear on both the wire and the brackets, indirectly contributing to lower friction. Using processes such as ion beam-assisted deposition, orthodontic archwires themselves can be textured with nanoscale patterns or columns to minimize binding and to reduce friction with bracket materials.

In summary, nanomaterials may enhance sliding mechanics between archwire and bracket interfaces through several mechanisms—improved surface smoothness, lubrication, altered textures, and precision manufacturing—that ultimately reduce binding and friction. Improved sliding facilitates better OTM. Advancements in nanocoatings and nanocomposite materials have been pivotal in achieving these improvements, although ongoing research is essential to further enhance these technologies and ensure their safety and efficacy.

### NPs enhanced corrosion resistance and improved biocompatibility

The corrosion of intraoral devices is a serious clinical concern. Indeed, numerous studies have documented the release of toxic ions from orthodontic appliances. New conventional stainless steel, recycled, and even nickel-free orthodontic brackets can release nickel [101] and chromium ions [102]. Likewise, an in situ evaluation revealed that silver solder used in orthodontics can lead to the release of copper ions [102]. Furthermore, the release of chromium from new stainless steel, recycled, and nickel-free orthodontic brackets has also been well-established in the literature [103]. Always associated with metallic ion release into the oral cavity, corrosion can be intensified by dental plaque accumulation and/or mechanical factors such as friction and fatigue stress. Several important consequences of this undesirable degradation may arise, namely enamel discoloration and demineralization, hypersensitivity, inflammation, local pain, device failure, and, in more severe cases, toxicity [104].

The need to modify orthodontic alloys is a currently recognized high-priority requirement. Research directions aim to (i) adjust the alloy bulk composition by using new and advanced manufacturing processes; or (ii) develop surface modifications that take advantage of the excellent mechanical properties of the bulk composition. The composition and microstructure of the surface can be altered by using chemical or physical methods, either by treatment or coating deposition. Surface modification and coating are attracting increasing attention, because it does not change the original properties of the substrate while conferring corrosion resistance.

Nanomaterials can be used to create protective coatings on orthodontic appliances such as braces and wires. These coatings are much thinner and more uniform than traditional coatings, and provide a more effective barrier against corrosive elements such as saliva and food. By enhancing the surface characteristics of orthodontic appliances with NPs, the biocompatibility of these materials can be improved. This, in turn, reduces the risk of adverse reactions, for example, by reducing inflammation in patients with metal allergies or sensitivities [104], improving biocompatibility, and enhancing patient comfort.

#### Metal/metal oxide nanomaterial coatings

The favorable physiochemical properties of TiO_2_ coatings should improve corrosion resistance [105]. TiO_2_ thin films exhibit ceramic-based structures, which have low electrical conductivity and lower charge transport, thus improving electrochemical barrier properties. Therefore, lowering the electron conductivity can impede electrochemical processes and thus improve the corrosion resistance of TiO_2_-coated devices. In general, coating materials should completely separate base materials from corrosive media to confer protection and robust corrosion resistance. Electrochemical reactions do not occur at the coating/substrate interface. TiO_2_ NP thin films were prepared by magnetron sputtering. The resultant coating surface was flat and compact and demonstrated good sealing performance, which can prevent contact between corrosive media and substrate materials. N-TiO_2_ coating can improve corrosion resistance more effectively than pure TiO_2_ coating [106, 107].

ZrO_2_ NPs occluded gaps in acrylic coatings through which corrosive ions and molecules could have penetrated, and hence delayed steel corrosion [108]. A relatively denser pore structure with uniform distribution morphologies of ZrO_2_/ZnO/TiO_2_ nanocomposite coating hindered the penetration of simulated body fluid and artificial saliva through the coating layer on a stainless steel substrate. The high durability and compactness of ZrO_2_/ZnO/TiO_2_ nanocomposite coating on stainless steel have conferred excellent corrosion protection in simulated body fluid and artificial saliva [109].

Among metal/metal oxide nanomaterials, ZnO-NPs possess a significant advantage in conferring corrosion resistance. The addition of a porous manganese-substituted hydroxyapatite coating to a ZnO-NP-coated 316L stainless steel alloy improved corrosion resistance, mechanical strength, and biological properties [110]. ZnO-NPs prepared on Ni–Ti wires not only inhibited *S. mutans*, but also lowered friction and improved the corrosion resistance of the substrate [96].

#### Graphene-based coatings

NiTi alloy substrates coated with either GO or GO/Ag nanocomposites exhibited enhanced corrosion resistance, reduced corrosion rates, and increased protection efficiency compared to uncoated NiTi alloy. The biocompatibility of the coated NiTi alloy was confirmed through the use of human pulp fibroblasts, which expressed elevated levels of IL-6 and IL-8 [111].

The application of various concentrations of GO coatings decreased the corrosion susceptibility of NiTi alloy in synthetic saliva, and also improved lubricity and *S. mutans* inhibition. Insufficient corrosion and friction resistances were observed in the coating when the concentrations of GO were either low or excessively high [112, 113].

The application of a small-sized GO/Ag NP coating to a NiTi alloy lowered the coefficient of friction to 0.1, conferred a tenfold decrease in corrosion current density, and reduced the presence of corrosive ions. However, a coating containing large-sized GO/Ag NPs brought only limited improvements of friction and corrosion resistance [114]. Both coatings were biocompatible with L929 cells, attributed primarily to the coating materials’ high biocompatibility at low concentrations. Additionally, corrosion resistance conferred by coatings prevents the precipitation of toxic ions, thereby enhancing biocompatibility.

A series of self-assembling polydopamine (PDA)-GO nanocoatings were applied to representative NiTi archwires. Coating morphology, chemical structure, and multifunctional performance were adaptable by changing the PDA/GO ratio. The optimized PDA–GO coating featured uniform and dense characteristics that increased the diffusion path of corrosive media and inhibited the dissolution of Ni in NiTi alloy. Furthermore, the surface structure and inherent characteristics of PDA–GO conferred antibacterial activity against *Streptococcus mutans* [115].

#### Polymeric coatings

Epoxy resin coatings significantly increased corrosion resistance [116, 117] and decreased nickel ion release [117] of NiTi archwire in artificial saliva. Additionally, a double-blind randomized clinical trial revealed that an epoxy coating reduced nickel ion discharge [118]. PTFE conferred a higher corrosion resistance than epoxy resin [117]. PTFE-coated NiTi archwires corroded ten times less quickly than untreated NiTi substrates [119]. On the other hand, PFTE-coated wire induced cytotoxicity in 36% of fibroblasts in an in vitro assay, which corresponded to slight cytotoxicity [120]. Therefore, selecting this material requires great thought. Epoxy resins are usually a preferable option if a high degree of biocompatibility is needed. A study recently used a mussel-inspired technique to encapsulate PTFE NPs in a sol–gel matrix and dip-coat them onto 316L stainless steel before the deposition of Ag NPs. Because of its potent antibacterial and anticorrosion qualities, the Ag NP/PTFE coating produced in this manner may be suitable for application to metal implant surfaces [121]. Clinical trials must be conducted on this coating as it has not yet been used in orthodontics.

Nanocoatings on orthodontic devices have the potential to mitigate the issue of ion release commonly associated with conventional metal braces. This is of utmost importance due to the potential consequences of metal ion release, such as toxicity, hypersensitivity reactions, tissue discoloration, and other adverse reactions. Consequently, thorough biocompatibility testing of modified materials is imperative to ensure safety and to rule out adverse reactions. Furthermore, corrosion resistance testing of nanomodified materials in simulated oral environments is essential. Such testing should address the corrosive effects of salivary pH fluctuations, bacterial colonization, and other environmental stressors. In the pursuit of enhancing corrosion resistance, the preservation of mechanical properties, antibacterial activity, and biocompatibility is imperative. The incorporation of NPs has the potential to modify the mechanical characteristics, including ductility and hardness, of orthodontic steel wires and brackets. Therefore, it is crucial to strike a suitable equilibrium that confers adequate corrosion resistance while preserving the mechanical properties necessary to accommodate the necessary orthodontic forces.

#### Strengths and limitations

This study addressed clinically relevant questions with significant implications for orthodontic treatment outcomes. Strengths include a methodology that references PRISMA guidelines. However, this review has limitations that must be acknowledged. Variability in study methods and potential biases among the 28 selected articles may have impacted our conclusions. Moreover, inconsistencies in measurement methods and definitions may have impeded data synthesis. Despite an extensive literature search, relevant studies may have been overlooked. Publication bias could lead to an overestimation of the benefits of nanotechnology. Future research should aim to standardize methods and broaden outcome measures to improve reliability and comparability. Despite these limitations, this review provides crucial insights into the potential of nanotechnology to advance orthodontic practice.

## Conclusion and future prospects

Nanotechnology offers considerable potential for the advancement of orthodontic treatment, presenting advantages such as improved materials, expedited tooth displacement, and enhanced oral health. As the field of nanotechnology progresses, it has the potential to significantly enhance the efficiency, efficacy, and patient experience of orthodontic care. Nevertheless, continued research and clinical trials are imperative to address safety concerns and to fully comprehend the ramifications of the application of nanotechnology to orthodontics. In the future, intelligent biocompatible materials and brackets will likely become integral components of the orthodontic armamentarium. Prominent areas of future research in biomedical engineering include the utilization of nanotechnology for device fabrication, the ongoing advancement of nanostructured and biomimetic materials, and the application of tissue engineering principles for both hard and soft tissues [122].

However, the transition of nanotechnology and nanomaterials from the laboratory to the clinic is hindered by significant challenges. Specifically, the long-term biocompatibility and safety of nanomaterials in the oral environment must be thoroughly investigated before their adoption can be considered. Despite the potential benefits of incorporating NPs, such as enhanced corrosion resistance, protection of moisture-sensitive components, and prevention of nickel release, it is crucial to evaluate the safety of the long-term intraoral retention of these materials. The oral cavity encompasses a complex milieu of digestive enzymes, fluctuating pH levels, a diverse microbiome, and mechanical stresses. Consequently, NPs may interact with oral tissues in unforeseen manners and may accumulate over time, potentially leading to toxic levels. To fully comprehend any previously unidentified adverse effects, long-term clinical trials and systemic studies are imperative. The evaluation of issues such as hypersensitivity, DNA damage, the accumulation of histopathologic changes, bacterial binding, and disruption of cell physiology is necessary. It should be noted that safety data for short-term usage may not adequately predict long-term outcomes.

Additionally, the utilization of nanotechnology in orthodontics may lead to increased treatment costs, thereby affecting accessibility. Although nanostructured materials may enhance the properties of components such as orthodontic brackets and wires, the associated manufacturing techniques significantly raise production expenses. Given the already considerable expense of orthodontic hardware, the incorporation of nanoinfused enhancements will inevitably contribute to cost increases. The biodegradation of nanomaterials poses a significant challenge due to the intricate control required. The pharmacokinetics of nanomaterials are heavily influenced by factors such as their size, shape, and surface chemistry. Achieving a consistent and uniform production of nanomaterials remains a formidable obstacle.

## Data Availability

No datasets were generated or analyzed during the current study.

## Abbreviations

Ag: Silver
BMSCs: Bone marrow mesenchymal stem cells
Cu: Copper
CuO: Copper oxide
ESR: Electron spin resonance
GFSs: Growth factor-loaded triphasic scaffolds
GO: Graphene oxide
LIPUS: Low-intensity pulsed ultrasound
MgO: Magnesium oxide
NEMS: Nanoelectromechanical systems
NO: Nitric oxide
NPs: Nanoparticles
OTM: Orthodontic tooth movement
PDA: Polydopamine
PDL: Periodontal ligament
ROS: Reactive oxygen species
TiO_2_: Titanium dioxide
WSLs: White spot lesions
ZnO: Zinc oxide
